# A case report of an unconventional chronic lead poisoning resulting from daily eyelid application of kohl

**DOI:** 10.1016/j.plabm.2025.e00517

**Published:** 2025-12-22

**Authors:** Charlène Aïn, Antoine Baudriller, Olivier Mathieu, Yoann Cazaubon

**Affiliations:** a Service of Pharmacology and Toxicology, University Hospital of Montpellier, Montpellier, France; b HydroSciences Montpellier (HSM), UMR5151, University of Montpellier, Montpellier, France; c Pathogenesis and Control of Chronic and Emerging Infections (PCCEI), Inserm, University of Montpellier, University of the Antilles, Montpellier, France

**Keywords:** Kohl, Cosmetic, Lead, Chronic poisoning, Neurotoxicity

## Abstract

**Introduction:**

Lead can be found everywhere in the environment and continues to be a public health concern. Lead exposure through kohl is a well-identified problem. In adults, the few reported cases show that this exposure is underestimated. The health consequences of chronic exposure are not well known.

**Case report:**

a 63-year-old woman contacted the Department of Medical Pharmacology and Toxicology to report suffering from numerous neurological disorders. She mentioned having previously undergone toxicological assessments showing abnormally high levels of lead and mercury about three years ago. Several lead tests revealed active lead exposure but at levels considered subtoxic, 170–198 μg/L. Subsequently, several interviews with an internist and a toxicologist allowed the identification of the contamination source: kohl. After discontinuing the use of kohl, it took a year for lead concentrations to drop below 50 μg/L. Apart from neurological symptoms, neither renal function nor hematopoiesis was affected.

**Discussion:**

This is the first case report describing the effects of chronic lead poisoning in adults over a period of more than 40 years. It is crucial to raise awareness among populations about daily using traditional kohls and to avoid these products due to their manufacturing process using galena.

## 1 Introduction

Lead is the cause of chronic intoxication arising from occupational, accidental or from less conventional sources of exposure such as cosmetic use. Kohl is a powder used for centuries for eye makeup in some countries. Traditionally, kohl is made from certain minerals, including antimony and lead. Its composition varies according to the region of the world and some kohls contain up to 95 % lead [1]. The FDA warns about the risks associated with the use of these traditional cosmetics, known by various names such as kohl, kajal, al-kahal, surma, tiro, tozali or kwalli. The use of lead-containing kohl can result in lead exposure, which can be toxic to health. People chronically exposed to lead can develop a wide range of symptoms, with severity often correlated with blood lead levels (BLLs), even there is no threshold effect linked to hearing loss and mental retardation in child. In adult, if subtoxic impregnation (threshold for antidote administration 300 μg/L) is mainly associated to risks on fertility in males, lead is foetotoxic and can cause complications in pregnancy (pregnancy-related hypertension, abortion or premature delivery, intra-uterine growth retardation and low birth weight, cognitive problems in children). Symptoms are often frustrating even for levels above 400 μg/L with asthenia and abdominal discomfort, nephrological and neurological signs often predominate. Numerous effects of lead poisoning have been reported in the literature, showing a polymorphic effects on various human body systems. Indeed, lead is distributed in different compartments, including red blood cells, soft tissues (liver, kidneys, lungs) and the skeleton [2]. Its elimination is mainly urinary, which explains its nephrotoxic nature. Moreover, lead is a biocumulative toxicant where the absorbed dose is much higher than the eliminated dose, complicating detoxification treatments. Herein, we report the case of a 63-year-old woman who suffered from neurological disorders for over 40 years caused by unconventional chronic lead poisoning.

## 2 Case report

In a context of diagnostic wandering, a 63-year-old woman contacted the Department of Medical Pharmacology and Toxicology to report suffering from numerous neurological disorders. She mentioned having previously undergone toxicological assessments showing abnormally high levels of lead and mercury about 3 years ago. Since then, no follow-up actions had been taken, and her neurological symptoms persisted. She was subsequently referred to the Department of Internal Medicine.

The patient's medical history began in 2017, when she was placed on sick leave due to a major depressive syndrome accompanied by months of asthenia. In May 2018, a metal/metalloid screening of her urine revealed abnormally high concentrations of lead (46.6 μg/g creatinine, reference value: <5) and mercury (28.4 μg/g creatinine, reference value: <1). Following these results, she received six monthly intravenous chelation treatments with sodium dimercaptopropane sulfonate at home. Follow-up BLLs were measured in October 2018 and January 2021, showing levels of 72 μg/L and 83 μg/L, respectively. On the day of the internal medicine consultation in August 2021, the patient reported increasingly marked neurological disorders with memory disturbances, spatial disorientation, immediate memory issues, headaches, and nearly constant tingling in the fingers and face. A magnetic resonance imaging was performed, showing a normal distribution of the white and supratentorial gray matter with a normal appearance of the peri-cerebral spaces and ventricular structures; the clinical examination was normal. Additionally, biologic tests, including an autoimmune assessment, complete blood count, biochemical tests, and a BLL were prescribed. The blood count and serum electrolytes show no abnormalities; the patient's estimated glomerular filtration rate (eGFR) by CKD-EPI was >90 mL/min, and her hemoglobin (Hb) was 13.7 g/dL. However, her BLL was elevated at 170 μg/L, potentially explaining her neurological issues. A peripheral blood film was not performed, as repeated complete blood counts did not show anemia or other cytopenias.

A report is made to the poison control centre, and a request to investigate the source of environmental contamination is initiated by the Regional Health Agency. BLL tests on her two children (men aged 24 and 27) were conducted to know if they are affected by active lead exposure. The results showed BLLs within the normal population range in France: 18 and 24 μg/L [3]. Professional exposure is considered due to her profession as a dental surgeon, but a 4-year gap between her cessation of work and the elevated BLLs does not suggest this as the source. A subsequent blood lead test in January 2022 revealed a BLL of 198 μg/L, indicating ongoing lead contamination. In April 2022, a phone conversation between the toxicologist and the patient led to the suspicion that kohl might be the source of contamination. The patient, born in Rabat, Morocco, had developed a habit of applying kohl to her eye contours every morning and afternoon since the age of 20. Upon considering this hypothesis, she immediately ceased using kohl and submitted the four samples corresponded to products purchased during different visits to Morocco (1980, 1984, 2017 and 2021). Although the earliest vials were initially purchased decades earlier, they were kept at home and intermittently reused; at the time of the investigation, the patient alternated between available vials. Approximately two months after discontinuing kohl use, her BLL was retested and found to be 98 μg/L. The lead concentration decreased, suggesting kohl as the likely source of contamination. Both whole blood samples and kohls were analysed with inductively coupled plasma mass spectrometry (ICP-MS) by selecting the 208 isotope of lead. The kohl samples were digested before lead, mercury and cadmium ICP-MS quantification. All contained very high lead concentrations (67.7–79.3 %) confirming suspicions, whereas mercury and cadmium were below the limit of detection (Table 1).

**Table 1:** Characteristics of the four samples of kohl used by the patient.

Location and date of purchase	Lead content (%, mg/100mg)	Mercury/Cadmium content	Macroscopic aspect
*Marrakech* *1980*	79,3 %	< LOD	
*Casablanca* *1984*	74,3 %	< LOD	
*Marrakech* *2017*	67,7 %	< LOD	
*Casablanca* *2021*	75,1 %	< LOD	

LOD: Limit of detection.

**Fig. 1:**
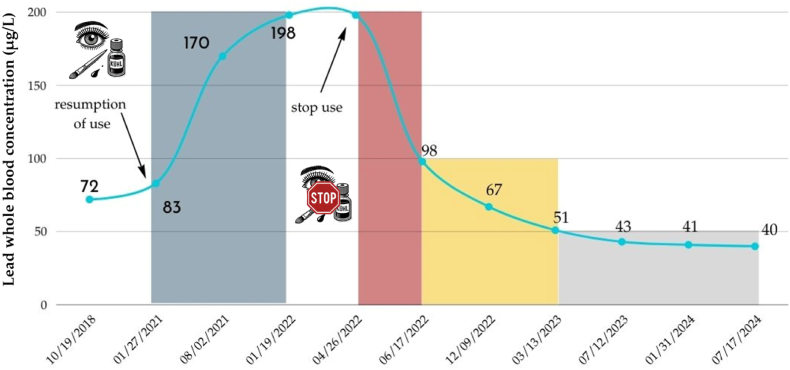
Time-dependent lead concentration in whole blood (μg/L). Absorption phase: Ka = 0.11 month^−1^. T_1/2Ka_ = 6 months; Elimination phase: Blood compartment: Ke_α_ = 0.014 day^−1^. T_1/2α_ = 50 days; Soft tissue compartment: Ke_β_ = 0.074 month^−1^. T_1/2β_ = 9.4 months; Bone compartment: Ke_γ_ = 0.071 year^−1^. T_1/2γ_ = 9.7 years. All constants have been calculated using a non-compartmental approach, by calculating the slopes of absorption and elimination.

She indicated having replaced the use of kohl with an organic cosmetic product between 2019 and early 2021. This information aligns with the observed increase in her blood lead level in August 2021 coinciding with the resumption of kohl use in the first quarter of 2021. Her BLL is monitored every 3–6 months. The results are presented in Fig. 1. All constants In March 2023, her complete blood count and serum electrolytes showed no abnormalities. Her eGFR by CKD-EPI was 93 mL/min/1.73m^2^, and her hemoglobin (Hb) level was 14.2 g/dL. In July 2023, a semi-quantitative urinary metal/metalloid screening, including lithium, beryllium, vanadium, chromium, manganese, cobalt, zinc, copper, arsenic, selenium, strontium, molybdenum, rhodium, palladium, cadmium, tin, antimony, barium, cerium, tungsten, platinum, mercury, thallium, lead, bismuth, thorium, and uranium, was undertaken to exclude any trace elements potentially responsible for her neurological disorders. The biological examination was normal. Plasma quantification of arsenic, antimony, and cadmium, as well as whole blood mercury concentrations showed no abnormal values. For that last heavy metal occupational exposition may be proposed on chronological considerations. In February 2024, the patient reported she was feeling better but continued to describe sudden episodes of intense fatigue.

## 3 Discussion

For the past fifty years, numerous studies have demonstrated that prolonged use of kohl is responsible for lead poisoning. Almost all of studies involved newborns and children [4]. Very few cases addressed this public health issue in adults [5,6].

Furthermore, the lead content in these cosmetic products can be extremely high, thereby increasing the risk of significant lead absorption [7]. In our case, the four samples brought by the patient had a shiny macroscopic appearance, which typically suggests the presence of galena and therefore lead. This leads us to suspect a probable high lead content, which will be confirmed by the assay.

Most of the reported cases in the literature concern children, with lead concentrations generally much higher than in adults. Only two case reports [5,6] and one case series of 20 regular kohl users [8] of chronic lead poisoning by khol were reported in adults between 1968 and 2025. For one patient, the concentrations were extremely high (4900 μg/L) leading to hospitalization with administration of the antidote [5] and for the other one [6], the concentrations and the frequency of exposition were comparable except for the duration (19 years versus >40 years in our case) and the symptoms. Regarding a series of 20 patients, the authors demonstrated that exposure to contaminated kohl led to a mean BLL that results in a statistically significant decrease in hemoglobin compared to the control group [8].

At the time of the consultation, the patient presented with neurological symptoms that significantly impacted her quality of life. The concentrations measured before discontinuing kohl exposure ranged from 170 to 198 μg/L, indicating ongoing contact with the source of contamination. These concentrations correspond to the lowest BLL at which adverse effects were observed by Schwartz et al.: 180 μg/L [9]. The estimated absorption slope shows a very intense resorption from application site. The elimination slopes of the three compartments, which characterise simplified toxicokinetics of lead, could be estimated after cessation of kohl application. Among the models studying the toxicokinetics of lead, the biological mean half-lives for lead are as follows: for the blood compartment, 25 days (adults, short-term exposure); for soft tissues (adults, short-term exposure), 40 days; for bone (labile, trabecular pool), 90 days; and for bone (cortical, stable pool), 10–20 years [2]. Regarding hemoglobin and renal function, neither has ever been impaired. This is consistent with the thresholds that predict the occurrence of such alterations. Chelation therapy with dimercaptosuccinic acid was not proposed given the recommended threshold of 300 μg/L [10]. It would have been considered if the patient had not improved over the years.

Our findings emphasize the importance of considering unconventional sources of lead exposure in patients presenting with chronic neurological symptoms. Little is known about the neurological impact of very long-term exposure at levels below those used for exposed workers. This case also highlights the need for public health interventions to raise awareness about the risks associated with traditional cosmetic products like kohl, which are still widely used in many cultures. In conclusion, two case reports and one case series describe the same type of intoxication but no one gives details about pharmacokinetics of lead in the human body such as absorption or elimination constants. This is the first case of chronic lead poisoning due to over forty years kohl use provides valuable insights into the pharmacokinetics, especially a very intense cutaneous resorption. It underscores the need for ongoing public health education and rigorous regulation of traditional cosmetics to prevent similar cases in the future.

## CRediT authorship contribution statement

**Charlène Aïn:** Writing – original draft, Investigation, Data curation. **Antoine Baudriller:** Writing – original draft, Methodology, Data curation. **Olivier Mathieu:** Writing – original draft, Validation, Formal analysis. **Yoann Cazaubon:** Writing – review & editing, Writing – original draft, Supervision, Methodology, Formal analysis, Data curation, Conceptualization.

## Ethical approval

In accordance with the guidelines of good clinical practice and in adherence with the requirements of the Declaration of Helsinki, this report was with permission and informed consent of the patient.

## Funding

This research received no external funding.

## Declaration of competing interest

The authors declare that they have no known competing financial interests or personal relationships that could have appeared to influence the work reported in this paper.

## Data Availability

The data that has been used is confidential.
